# Freelance information management agents: why information management is so hard on translational teams

**DOI:** 10.1017/cts.2023.616

**Published:** 2023-09-04

**Authors:** Jason Chladek, Patrick W. Kelly, Betsy Rolland

**Affiliations:** 1 Institute for Clinical and Translational Research, School of Medicine and Public Health, University of Wisconsin-Madison, Madison, WI, USA; 2 Social and Administrative Sciences Division, School of Pharmacy, University of Wisconsin-Madison, Madison, WI, USA; 3 Carbone Cancer Center, School of Medicine and Public Health, University of Wisconsin-Madison, Madison, WI, USA

**Keywords:** Information behaviors, information management, translational teams, clinical and translational research

## Abstract

**Introduction:**

To conduct high-quality, rigorous research, and advance scientific knowledge, Translational Teams (TTs) engage in information behaviors, including seeking, using, creating, sharing, storing, and retrieving information, in ways specific to the translational context. Currently, little is known about TTs’ approach to information management. This qualitative pilot study explored how TTs at the University of Wisconsin-Madison interact with information, as well as the scientific and organizational impact of their interactions.

**Methods:**

We conducted interviews with ten members of UW TTs. Interviews were transcribed and thematic analysis was conducted.

**Results:**

Four themes emerged: (1) TT members did not recognize the centrality of information or information behaviors to their scientific work; (2) TT members engaged in similar information behaviors and used similar tools across disciplines and topics; (3) TT members did not receive support or guidance from their institution in managing information; and (4) Individualized choices of TT members conflicted at the team level, causing confusion and increasing the potential for data and information loss. Acting as *freelance information management agents*, TT members made individualized decisions about what tools to use and how to use them, often in a piecemeal manner and without communicating these decisions to other team members.

**Conclusion:**

Research institutions should both encourage teams to discuss their information management approaches at the beginning of a project and provide leaders with training on how to have these conversations and what topics should be included. Additionally, institutions can provide researchers with guidelines for using software platforms to help mitigate information management challenges.

## Introduction

Translational research, a process that involves moving scientific discoveries from the bench to the clinic to the community in order to create sustainable improvements in society, requires a team-based approach [1,2]. Translational Teams (TTs) span a variety of both clinical and non-clinical specialties and are often interdisciplinary in nature, integrating perspectives from basic scientists, population scientists, clinical providers, community partners, and other stakeholders [3].

In order to conduct high-quality, rigorous research, and generate and advance scientific knowledge, TTs must engage with and manage various types of information that are specific to the translational context and environment in which they are working. Thinking specifically about the context of conducting Clinical and Translational Research (CTR), here we use the definition for scientific data put forward by Borgman et al. (2015): “Data are representations of observations, objects, or other entities used as evidence of phenomena for the purposes of research or scholarship” [4]. We can then think of *information* in scientific research as the human-generated digital objects of the work needed to generate, use, manage, and report those data. The use of information relies on TTs engaging in *information behaviors*, including seeking, using, creating, sharing, storing, and retrieving information [5]. The collection of information behaviors in which TTs engage constitutes their approach to *information management* through the use of tools and platforms.

Like most knowledge workers, TTs members are drowning in information [6]. A lack of guidance on how best to manage their information leaves researchers struggling to develop both individualized and team-based information behaviors that facilitate, rather than hinder, their scientific work. Universities provide a set of tools to all employees (e.g., word processing software, file sharing repositories), tools that were rarely developed with the specific work of scientific research in mind. Additionally, while numerous information behavior and management frameworks exist, they do not neatly fit the unique characteristics and processes of TTs, as their work often includes collaboration across institutional, disciplinary, and geographic boundaries and emphasizes the translation of findings from clinical environments to the public [7]. Further, these unique characteristics alone can exacerbate information management challenges.

To our knowledge, this is the first study to focus on the information behaviors of teams, either general teams or TTs more specifically. As a result of this gap, we know little about the types of information used by TTs or how TTs recognize, engage with, make decisions around, or manage information to accomplish translational research. This lack of guidance leaves each translational researcher and each TT to figure out their own approach in a vacuum. Structured and consistent approaches to information behaviors can support translational research efforts [8]; therefore, a better understanding of what provides structure and helps TTs perform information behaviors more effectively and efficiently can help us develop optimal approaches and tools to support TTs in translational research.

The goal of this pilot study was to explore how members of TTs at the University of Wisconsin-Madison interact with information and describe the scientific and organizational impact of their information behaviors. In doing so, we hope to highlight for those conducting and supporting CTR the role information plays in their research, their own information behaviors, and the impact those behaviors may have on their scientific work.

## Materials and methods

We conducted qualitative interviews with members of CTR teams employed by the University of Wisconsin-Madison. The UW-Madison Office of the Vice Chancellor for Research and Graduate Education – whose mission includes supporting multidisciplinary research centers and institutes – oversees $1.3 billion in annual research expenditures, putting UW-Madison among the top 10 in the nation among universities for volume of research. The Institute for Clinical and Translational Research (UW-ICTR) serves as the UW-Madison Clinical and Translational Sciences Award (CTSA), facilitating CTR across the university. The protocol for this study was deemed exempt by the University of Wisconsin IRB.

We operationalized TTs by identifying research labs affiliated with UW-ICTR. Individual TT members were further identified using research networks within the UW School of Medicine and Public Health and UW-ICTR’s databases of past training and funding awardees. We recruited one individual from each team, and potential participants received an email (Appendix A) from the research team describing the study and participation requirements. Participants were emailed in rounds of ten, including a mixture of Principal Investigators and/or Academic Faculty, Research Specialists or Scientists, Program Managers, and Postdoctoral Researchers or Graduate Students. The goal of this recruitment method was to include participants that represented as many of the 12 CTR *personas* identified by Gonzales et. al (2020) as possible [9].

**Figure 1.**
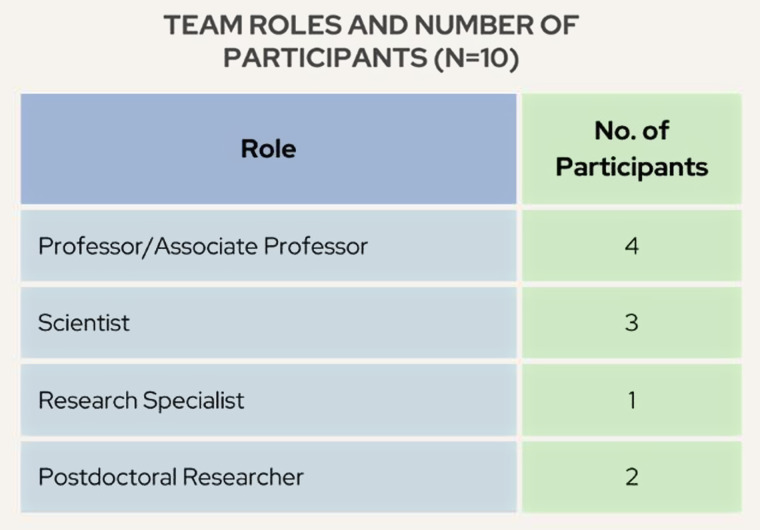
Number of participants by role on translational research team.

Ten participants were included in the final study, as outlined in Fig. 1. Participants represented ten different TTs and hailed from the School of Pharmacy, Department of Family Medicine and Community Health, Ophthalmology, Nutritional Science, Dermatology, Neurology, and Industrial and Systems Engineering. All participants were working on at least one grant-funded CTR project, and to our knowledge, none of the participants were working on overlapping projects. Interviews included semi-structured questions (Appendix B) related to the overall goal of their research projects and the activities and tools required to complete specific research tasks. Each participant was interviewed once for one hour by a member of the study team, either J.C. or B.R. All interviews took place via Zoom and were video and audio recorded.

The interviews were transcribed verbatim, and the participants were given pseudonyms. Two coders (J.C. and P.K) performed coding of interview transcripts and were supervised by the principal investigator (B.R.), who has experience in qualitative approaches. Coding was conducted via NVivo 12 software (released in 2018) [10]. The transcripts were coded across three rounds. First, the coders performed structural coding, which included coding the data according to each question in the interview guide. Structural coding allowed the researchers to compile responses for each question and identify similarities and differences among the responses. Second, transcripts were coded for barriers and facilitators under five domains constructed by the researchers (J.C. and P.K.): mental models; team structure, culture, and environment; project management; external factors; and tools. Third, transcripts were coded by research activity (e.g., exploring and defining research topics; building research support systems; holding team meetings; sharing results and transferring knowledge); these domains were constructed based on our literature review on the information behaviors of teams and a modified version of the “Information Seeking Behaviour Model” developed by Salaiegheh & Hayati (2009) [11]. Coders discussed and resolved coding discrepancies during weekly meetings. Finally, the coders created memos to merge the three rounds of coding and identify emergent themes. These themes were discussed and finalized with the research team, and the coders selected representative quotes for each theme.

## Results

Participants came from teams of varying sizes and worked on an array of clinical and non-clinical grant-funded projects across the translational spectrum. Three participants described projects focused on identifying biomarkers, while another cluster of participants described the implementation, dissemination, and evaluation of system-level interventions. Additionally, a majority of the participants reported working on more than one research project simultaneously, and these projects typically included team members from across different colleges and disciplines, as well as individuals in different career stages. Two participants described partnerships with other universities, and two additional participants discussed translational research projects that included key community partners. Cross-disciplinary work and collaboration with external or community partners presented additional information challenges.

**Figure 2.**
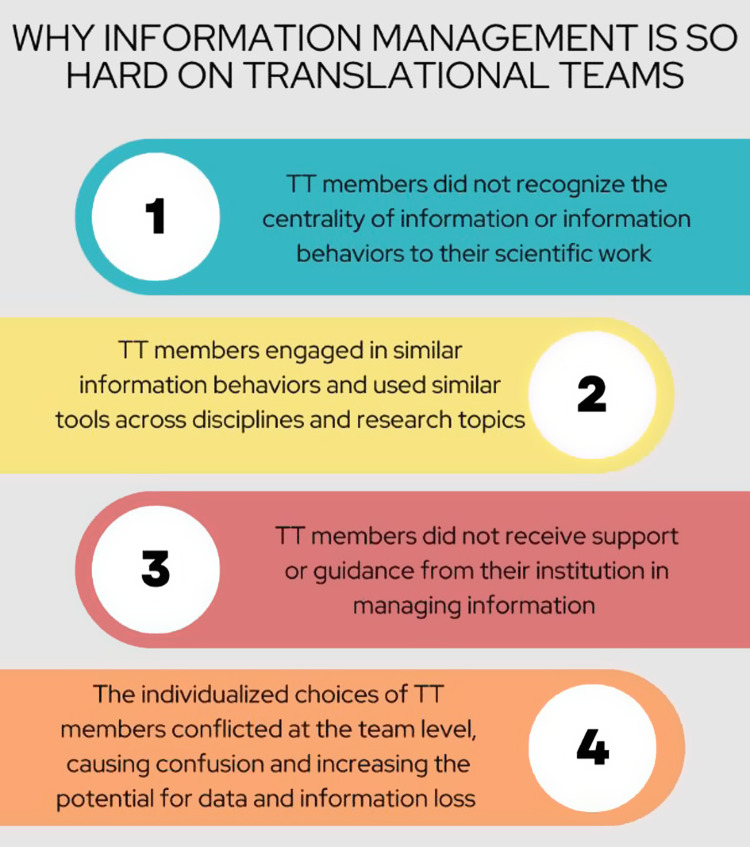
Emergent themes developed from interview data.

We identified the following four themes in our data (Fig. 2):

### Theme 1 – TT members did not recognize the centrality of information or information behaviors to their scientific work

Despite the fact that the conduct of CTR requires substantial interaction with and use of information, TT members did not recognize the significant impacts that information behaviors and management can have on the successful progress and translation of their work. Although our interview questions prompted the participants to recognize and discuss their use of information, most participants were initially surprised by these questions. Participants stated that they didn't explicitly think about how they were engaging with information or how information behaviors fit into the overall context of their scientific work. When asked about how her team stored and shared information, Katie said it was an “interesting question” because she and her team hadn't thought a lot about these behaviors (Katie, 135).

Additionally, because TT members did not recognize the centrality of information behaviors, they did not think about the consequences of their strategies or choices pertaining to these behaviors – especially concerning the tools and platforms used to store and share information. In regard to her team’s project, Katie explained, “Someone just started using PPT slides, so that’s when we started using PPT slides. And someone tried to share it through Google Drive or Box, and then we started using Box as a platform to share it.” She added, “I don't think we really talked about how we’re going to share it within the team. It just naturally happened,” (Katie, 135–8). Other participants in our study shared that they were more reactive in terms of determining how to share and store information, and improvising solutions on the fly as they presented themselves. Ben described this struggle as, “the things that keep me up at night that no one ever talks about when you’re talking about conducting research,” (Ben, 515–6).

### Theme 2 – TT members engaged in similar information behaviors and used similar tools across disciplines and research topics

**Figure 3.**
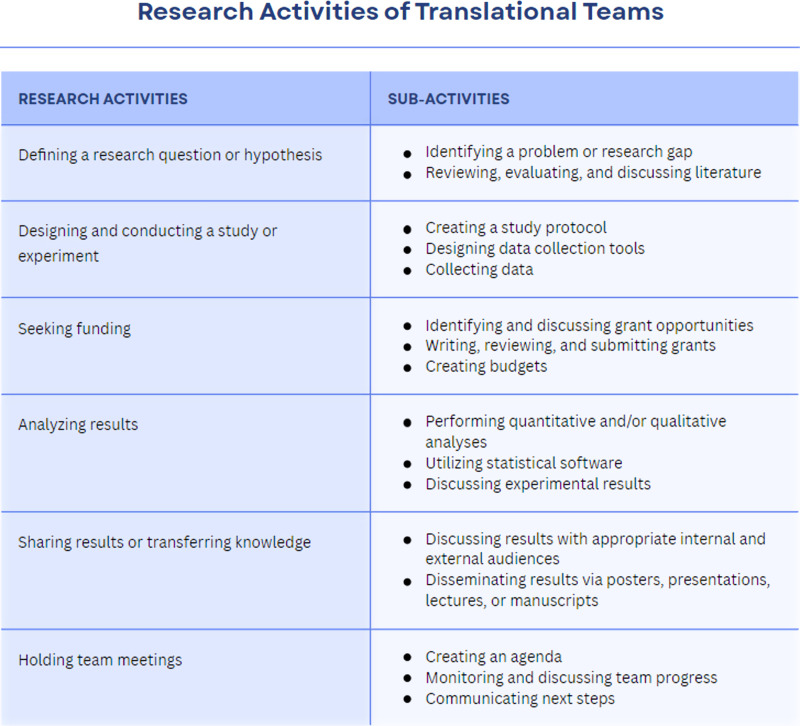
Scientific and organizational tasks and activities reported by participants.

Despite representing a wide range of roles and scientific disciplines, participants reported similar scientific and organizational tasks and activities related to their research projects [12]. As shown in Fig. 3, all of the sub-activities described by our participants fell within the research activity domains constructed during the third round of coding. While the work of individual TT members may look different at the granular level, zooming out, we saw that they were doing the same general types of information work in the conduct of translational research. In other words, in order to complete their scientific work, participants engaged in similar information behaviors – including sharing and storing information. Additionally, they used similar tools and platforms to carry out these information behaviors. Common tools and platforms included email, Box, OneDrive, Microsoft Teams, Microsoft Office, Webex, Google Docs, Research Drive, REDCap, and Zoom, all of which were provided as free, default, and approved software by UW-Madison.

### Theme 3 – TT members did not receive support or guidance from their institution in managing information

TTs reported receiving little to no support or guidance from their institution in managing their information. As noted above, UW-Madison provided a number of standard software packages for researchers to use; however, in the absence of evidence-based practices, the university provided no additional help. Further, the tools that were provided were rarely interoperable or customized to the needs of translational research. One PI, Steve, summarized it with, “Nobody tells you what tools you should use,” (Steve, 126). Alex, an Associate Scientist, discussed his struggle in finding the right platform for sharing information. Alex said, “I like the One Drive platform, but it is missing the security. Because of it, IT doesn't like it. But like, the way I can open directly on my computer from the One Drive, and I can change and add stuff, and it automatically saves it. For example, with Box, IT guys think it’s more secure. But then with the Box, like, you need to download, you need to work on your computer, and go back again. That’s the draw right there.” Acknowledging the need for collaboration, he added, “Like everybody has to directly reach that file, or whatever they’re working on, without any hassle. Work on it and leave it, you know. And then the next person can come and see it. That’s the important part, compatible and continuously updating and saving,” (Alex, 262–9). Alex’s comments highlighted how a lack of University guidance created difficult situations for TT members, forcing them to make tricky information management decisions on their own within parameters set by the university, which may or may not apply to their own projects.

Additionally, without guidance, individual TT members had to make sense of a confusing bureaucracy of University security standards. This mishmash of standards was especially a problem, our participants explained, when TTs were collaborating with outside partners or working with HIPAA-protected protected health information (PHI). Nancy said that setting up a secured research drive for storing both research data and information was a “lengthy process at the beginning.” She added, “Sometimes I just wish there was a more streamlined way of doing it, you know” (Nancy, 263–5). Another participant, Ben, added that getting secured server space was a “time consuming” process that required “jumping through hoops,” which took time away from scientific work. Moreover, he added: “There’s so many different approvals that you have to get to really do things right, and it’s more time consuming. Things take so much longer than, you know, they should by like 6 months, or maybe even more. So, I find that pretty frustrating,” (Ben, 545, 489, 544–7). He finished by saying, “I feel like I’m doing less and less science and more of just this type of… administration. The longer I’ve been here, the less and less science I’m actually doing” (Ben, 532–4). This sentiment from Ben highlighted how the lack of guidance from the university around information work had a direct impact on TT members’ productivity and scientific work.

Finally, another PI, Sarah, noticing how she saw her team’s information management as inextricably linked to its approach to communication, added that there was no guidance for how a TT leader should communicate with their team. Specifically, she said, “[Share] what [information]? With whom? How do you balance the commitment to participatory processes and a team approach with the need for efficiency?… How do you communicate in a modality that’s respectful, you know?… And when is it better to communicate with people individually versus in a group?” (Sarah, 344–358). This issue was further complicated when TTs collaborated with community partners, which was often encouraged or even required by the university or funding agencies. The participants described projects that included a number of community partners, including churches, senior living centers, health departments, and community pharmacies. However, there was no guidance on the best approaches for TTs to communicate with these partners.

### Theme 4 – The individualized choices of TT members conflicted at the team level, causing confusion and increasing the potential for data and information loss

As a result of the challenges described above, individual members often made different decisions than other members of their current and future teams, resulting in conflict at the team level (see *Toward a Translational Team Science Hierarchy of Needs: Exploring the Information Management Challenges of Team Science* [13] for more details). Alex, a Scientist, stated that, “Everyone kind of picks their own way to keep information about experiments [and] all that stuff,” (Alex, 172–3). Four participants explicitly expressed frustration over a lack of clear documentation for a team’s approach to information management. As Ben said, “I’d probably say the documentation isn't quite there because there’s also been times where I’m looking to find what a particular individual said on something specific,” and he wasn't able to do so (Ben, 410–1). Ben also noted that everyone on his team saved files according to their personal preference, which invariably created confusion. Nancy explained how confusing it was when she first joined her TT, as she had to navigate a Box folder structure she didn't create and whose organization was never explained to her. She struggled with how to label and store information, stating, “Like I can put it somewhere that kind of makes sense to me. But then if nobody else can find it, what good is it, you know?” (Nancy, 222–3).

The lack of recognition of the centrality of information to their scientific work meant that TTs rarely discussed how to manage that information. Without discussion about their individualized information behaviors and choices, TT members often ended up using different methods and different approaches to storing both information and data. These approaches were rarely documented or communicated to others on the TT. Nancy explained that because her team used multiple platforms, certain members would accidentally upload documents to the wrong platform. Likewise, Alex reflected on the difficulty of “trying to remember” where minor project details were saved or stored, a process often made worse by the absence of a clear documentation system (Alex, 176). Katie also added that using multiple platforms made information sharing more difficult, as it created more opportunities for information to get lost or misconstrued. She said, “But after going into the field one day, I realized, like, “Oh, the data from my previous person, she or he forgot to upload. Am I the only one who’s not able to find where it is?’ And I suspected, like, multiple reasons why before I approached that person because I wanted to make sure, like, it’s not me. So, the first reason I suspected was there are multiple platforms or systems that we’re using, and some people, especially the new data collectors, might not be aware that we need to upload to this system instead of that one or that kind of thing for specific types of data,” (Katie, 231–6).

## Discussion

In order to accomplish the necessary scientific and organizational tasks required to conduct translational research, TTs engage in key information behaviors, including seeking, using, creating, sharing, storing, and retrieving information. TTs support these behaviors using primarily university-provided tools and platforms. However, our data show that TT members do not recognize the importance of information itself or their behaviors, let alone the need for a cohesive approach to information management, until they are faced with a challenge or conflict.

In essence, translational researchers are forced to act as *freelance information management agents:* they make individualized decisions about what tools to use and how to use them, often in a piecemeal and reactive manner and without communicating these decisions to other team members. These individual choices then impact how the entire team approaches information management. A key challenge is that while translational researchers are implicitly expected to seamlessly manage the overwhelming amount of information required to conduct translational research, they are rarely trained to do so effectively and efficiently, resulting in challenges and conflicts for the entire team. Additionally, a lack of communication about information management decisions conflicts with “best practices” for team science. Previous work has demonstrated that communication, including discussions on logistical strategies, is critical to successful team functioning and strong research processes [14].

The lack of formal training is compounded by a university structure that makes it difficult for researchers to navigate administrative processes and a lack of recommendations for how teams, in general, should approach information management. This lack of recommendations is not surprising given how little we know about the information behaviors of TTs, the gap our study was designed to begin to address. Security restrictions from the university, especially around PHI, add another layer of complexity to information sharing with external individuals, teams, or organizations.

The information management challenges described here are a problem for the field of CTR because they can result in the loss of key information and data, wasted time and effort, and delays in achieving translation of important scientific findings. When elaborating on the difficulties of information management, participants described themselves as feeling overwhelmed and frustrated and highlighted the sheer amount of time it takes to address problems related to information management. The complexities of information management, combined with a lack of best practices, mean that grant funds are wasted on time spent navigating information management conflicts. As PI Ben said, he felt like he was doing “less and less science” every day (Ben, 532–3).

TT members can start to address some of the challenges described here by learning to recognize the information they are using, acknowledging the importance of this information to their research efforts, and having a conversation about how information management will work on their TT. These conversations should include not only a discussion of internal information management strategies, but also a plan for collaboration with external and community partners when appropriate. TTs could also utilize existing evidence-based interventions to support these conversations. For example, the Collaboration Planning sessions offered by UW-ICTR’s Team Science Core help TTs proactively think about the types of information they will use, determine documentation strategies, and plan how team members will be trained on these processes [15].

### Limitations

The results of this study are both exploratory and descriptive in nature, and some limitations exist. First, the number of participants interviewed was small and represented just four categories of TT members. While we felt we achieved saturation with ten participants using the existing instrument, we recognize the limited sample size and look forward to expanding to more participants in the future. Second, while many of the Principal Investigators took on the responsibilities of Program Managers, our study did not include any TT members with this title. We look forward to including Program Managers in future work. Third, the study took place at one large Midwestern research institution with an active CTSA and Team Science program; as such, the results may not generalize to TT members in other environments. However, as the first study of the information behaviors of TTs of which we are aware, we believe these results create a starting place for learning more about this topic.

## Conclusion

Information management is foundational to the conduct of CTR. However, while information is central to the scientific and organizational work of TTs, team members rarely recognize that centrality, lack guidance on how to effectively manage that information, and devote little effort to planning and aligning their information behaviors with those of their teammates. Each TT member makes individualized choices regarding information management, yet rarely communicates with their collaborators regarding these choices. This lack of attention to information leads to team-level challenges that hinder the progression of scientific work.

We believe that a minimal investment by research institutions in providing researchers with even modest guidelines for using key software platforms, both individually and as teams, coupled with encouraging teams to discuss their information management approaches at the beginning of a project, could go a long way in mitigating some of the challenges of information management. Further, providing training to teams, especially team leaders who may be unsure of how to have such conversations or those working with external or community partners, could focus on how to have information management conversations and what topics to cover. As discussed above, evidence-based interventions such as Collaboration Planning can also support these conversations.

Clearly, much more research is needed to better understand how to address the challenges outlined here, as well as solutions to mitigate those challenges, and we have recently received funding to expand this pilot study. Additional research may point toward the development of evidence-based pieces of training, recommendations, or tools for TTs. These findings could also inform the development of a comprehensive information behavior and management framework that considers the unique characteristics of TTs and their work. Lastly, future research may also focus on how institutions can better support TTs and provide “plug and play” solutions for a team’s information management strategy. All of these options could represent low-burden opportunities for TTs to improve information management approaches and, as a result, enhance research translation.

## Supporting information

Chladek et al. supplementary material 1 Chladek et al. supplementary material

Chladek et al. supplementary material 2 Chladek et al. supplementary material

## Supplementary material

The supplementary material for this article can be found at https://doi.org/10.1017/cts.2023.616.
